# On the Prediction of Lattice Energy with the Fukui Potential: Some Supports on Hardness Maximization in Inorganic Solids

**DOI:** 10.1021/acs.jpca.1c09898

**Published:** 2022-06-29

**Authors:** Savaş Kaya, Andrés Robles-Navarro, Erica Mejía, Tatiana Gómez, Carlos Cardenas

**Affiliations:** † Health Services Vocational School, Department of Pharmacy, Sivas Cumhuriyet University, Sivas58140, Turkey; ‡ Departamento de Física, Facultad de Ciencias, Universidad de Chile, Las Palmeras 3425, Santiago Casilla653, Chile; § Facultad de Ingeniería−(Medellin-Colombia), Institución Universitaria Pascual Bravo, Medellín050025, Colombia; ∥ Theoretical and Computational Chemistry Center, Institute of Applied Chemical Sciences, Faculty of Engineering, Universidad Autonoma de Chile, Santiago9170124, Chile; ⊥ Centro para el Desarrollo de la Nanociencia y la Nanotecnología (CEDENNA), Avda. Ecuador 3493, Santiago9170124, Chile

## Abstract

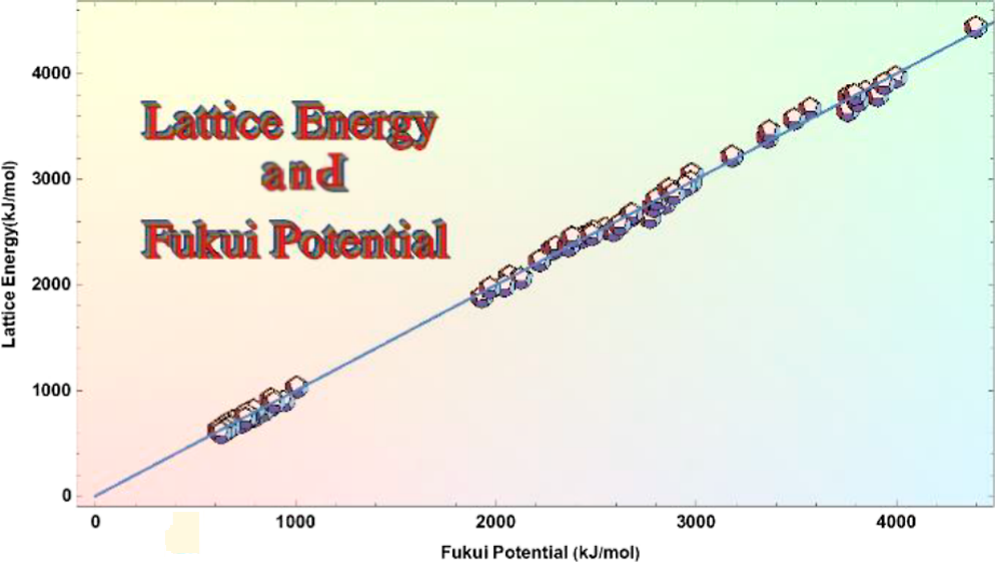

Using perturbation theory within the framework of conceptual density functional theory, we derive a lower bound for the lattice energy of the ionic solids. The main element of the lower bound is the Fukui potential in the nuclei of the molecule corresponding to the unit formula of the solid. Thus, we propose a model to calculate the lattice energy in terms of the Fukui potential. Our method, which is extremely simple, performs well as other methods using the crystal structure information of alkali halide solids. The method proposed here correlates surprisingly well with the experimental data on the lattice energy of a diverse series of solids having even a non-negligible covalent characteristic. Finally, the validity of the maximum hardness principle (MHP) is assessed, showing that in this case, the MHP is limited.

## 1 Introduction

Lattice energy is an important parameter of solid-state chemistry and physics as it provides insights about the thermodynamics and chemical reactivity and stability of inorganic ionic crystals.^1^ Lattice energy is defined as the energy required to decompose a mole of the solid in its gaseous ions.^2^ The lattice energy cannot be determined directly from an experiment because it is not possible to dissociate an inorganic solid into its gaseous ions.^3,4^ Indirect experimental procedures and some useful theoretical methodologies to compute the lattice energies of inorganic and organic solids are available in the literature. Therefore, its determination is based on indirect experimental quantities and thermodynamic cycles such as the Born–Haber–Fajans cycle.^5^ This cycle uses experimental data that can be determined very accurately, such as ionization energy, electron affinity, bond dissociation energy, atomization, and formation enthalpies. Therefore, the values of the lattice energy obtained through the Born–Haber–Fajans are normally accepted as the experimental ones. Lattice energy can also be estimated via quantum mechanical calculations, as well as computational thermodynamic data.^6^ However, quantum mechanical calculations can be applied, in general, only to simple systems. Therefore, phenomenological models that allow a quick evaluation of the lattice energy of solids for which thermodynamic information is not fully available to determine the lattice energy are highly valued. For instance, lattice energy can also be modeled from classical lattice electrostatic energy, for which the knowledge of the lattice of the crystal and effective ionic radii is needed. The first works in this direction were carried out by Born–Lande^7^ and Born–Mayer,^8^ who proposed equations for the lattice energy of inorganic ionic crystals. Later, Kapustinskii^9^ proposed a generalization of those original works so that his equation can be applied to the ionic systems whose lattice types are unknown. The Kapustinskii equation reads
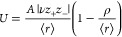
1where *z*_+_ and *z*_–_ stand for the integer charges on the cation and anion of the crystal, respectively. ν is the number of ions per formula unit. ρ is a parameter of the model known as the compressibility constant, whose fitted value is ρ = 0.0345 nm.  is the sum of the ionic or thermochemical radii of ions in the crystal. *A* is a constant fitted to 121.4 kJ mol^–1^ nm. Born–Lande, Born–Mayer, and Kapustinskii equations assume that the crystals are 100% ionic. Hence, eq 1 performs worse whose covalent characteristic cannot be neglected.

The Kapustinskii equation was originally derived for binary ionic solids. After more than 6 decades, Glasser^10^ noticed that the Kapustinskii equation can be generalized to compute the lattice energies of both simple and complex systems if the ionic strength (*I*), instead of , is used
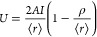
2

The relation between the ionic strength, (*I*), and the number of ions (*n*_*i*_) having integer charge *z*_*i*_ in the crystal is . In eq 2,  stands for the weighted mean cation–anion radii sum.

In 2003, Zhang and co-workers^11^ introduced an empirical methodology to predict the lattice energies of inorganic ionic crystals based on chemical bond theory. In the Zhang approach, the total lattice energy is split into ionic (*U*_i_) and covalent (*U*_c_) contributions
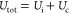
3

The ionic contribution to the lattice energy of single-bond binary ionic crystals with formula *A*_*m*_*B*_*n*_ can be given
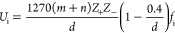
4

In eq 4, *Z*_+_ is the charge on the cation and *Z*_–_ is determined from neutrality, . Equation 4 also includes the bond length (*d*) and the fractional ionicity (*f*_i_). The covalent contribution to the lattice energy is related to the fractional covalency (*f*_c_) and charge on the cation
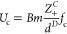
5

The fitted values of *B*, *C*, and *D* are 2100, 1.64, and 0.75 kJ/mol^–1^, respectively.

In volume-based thermodynamics (VBT), a technique introduced by Jenkins and Glasser,^12−17^ the thermodynamic parameters are correlated with the molar volume (*V*_m_). For the lattice energy of inorganic ionic crystals, the authors proposed the following correlation
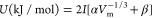
6

Here is again the ionic strength (*I*) and α and β are the coefficients that depend on the stoichiometry of the crystal. Equation 6 provides results very close to experimental data for ionic systems with the lattice energy less than 5000 kJ/mol.

As a large lattice energy implies a large thermodynamic stability, eq 6 suggests that a small molar volume is also a measure of stability. Now, the maximum hardness principle (MHP)^18−22^ states that “there seems to be a rule of nature that molecules arrange themselves so as to be as hard as possible”. Hence, if the MHP applies to solids and not only to molecules, there should also be a relationship between the chemical hardness,^23−25^ η, and the lattice energy. Indeed, Kaya and Kaya^3,26^ investigated the relationship between chemical hardness and lattice energy of inorganic ionic crystals and derived the following equation
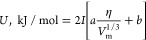
7where *a* and *b* are the coefficients that depend on the stoichiometry of the crystal and η is the chemical hardness of the molecule in the formula unit of the solid. For instance, in sodium chloride, η would be the hardness of the diatomic molecule NaCl.

Chemical hardness (η)^23−25^ is a measure of the resistance against electron cloud polarization or deformation of a chemical system. This concept was introduced along with the proposition of the HSAB principle^19,27−32^ which states that “*all other things being equal, hard acids prefer binding to hard bases and soft acids prefer binding to soft bases*”. It is clear from here that chemical hardness is closely related to the stability and reactivity of chemical systems. Although Pearson’s original definition of η did not offer a quantitative scale of hardness, further on, he and Parr proposed a mathematical definition of hardness within what is now known as conceptual density functional theory (CDFT):^33−39^ the chemical hardness is the second derivative of energy, *E*, with respect to the number of electrons, *N*, which equals the first derivative of the chemical potential with respect to *N*(19)
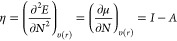
8where *I* and *A* stand for ground-state vertical ionization energy and ground-state vertical electron affinity, respectively.

In this paper, we will elaborate on the link between lattice energy and known CDFT reactivity descriptors. In particular, we will show that the interaction energy between ions forming a crystal suggests a strong link between the Fukui potential^40−43^ and the lattice energy.

This paper is organized as follows: in the Theory section, we will derive a lower bound for the lattice energy which depends only on the Fukui potential at the atomic position and which allows us to propose a simple expression for the lattice energy (eq 16). Then, in the Results and Discussionsection, we will show the performance of our expression by checking it against the experimental data and other expressions for the lattice energy presented in this introduction.

## 2 Theory

Lattice energy is defined as the energy that takes to dissociate an ionic solid into its atomic ions. Let us for a moment think in a diatomic molecule MX, where M stands for the metal (Na^+^) and X for the non-metal (Cl^–^). Let us assume that in the dissociation of the molecule into its ions
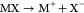
9there is no electron transfer among them. That is, the M–X bond is strongly ionic. Hence, the dissociation energy, Δ*E*, can be approximated with perturbation theory as far as the electronic states of molecules is not degenerate^44^
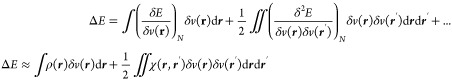
10where  is the change of the external potential acting on the electrons,  is the electron density, and  is the linear response function.^45^ In a strongly ionic bond, the valence electron density is well localized around the non-metal anion. Hence,  in the dissociation is approximated by
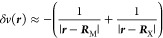
11

Equation 11 also applies to ionic solid but it has been multiplied by the Madelung constant. For the sake of simplicity, we will obvious that constant thereof. Note that replacing eq 11 in eq 10 leads to the conclusion that the first term is an electrostatic contribution, Δ*E*_elc_, while the second is a polarization term that explains the electron rearrangement upon dissociation. For strongly ionic solids, one expects the electrostatic contribution to depend mostly on the lattice parameter.

Now, if electron correlation is neglected and noting that the largest contribution to χ is from the frontier orbitals Φ_HUMO_ and Φ_LUMO_
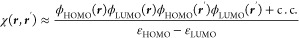
12

Using the Cauchy inequality and following the procedure in Eqs 135 to 138 in the work by Ayers,^42^ one shows that
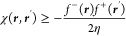
13where  are the Fukui functions^46,47^ for accepting and donating electrons. Replacing eqs 11 and 33 in eq 10, and assuming that the HOMO orbital is well localized around the metal and the LUMO around the non-metal, one gets
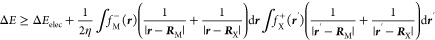
14

Because  is well localized around  and  is around , one can neglect the second and first terms in the first and second integrals, respectively.
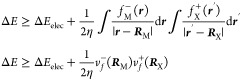
15where  is the Fukui potential^48,49^ at the atomic position **R**_**α**_.

Cardenas and co-workers^50,51^ showed that the Fukui potential at nuclear positions is a measure of the resistance of an atom to change its state of charge. A similar interpretation is valid for chemical hardness. It is apparent from this that hardness and the Fukui potential are related parameters. Cardenas^52^ showed that in the cases of atoms, the Fukui potential at the nucleus equals the hardness of the atoms and that in molecules it correlates well and even outperform other descriptors of local hardness. The difference between chemical hardness and Fukui potential is that chemical hardness is based on the changes on the number of the electrons while the Fukui potential is based on the changes on the atomic number, which is what is formally known as an alchemical derivative.^53−56^ Chattaraj, Cedillo, and Parr proposed^57^ another link between Fukui potential and hardness, and they argued that chemical hardness can be defined as the Fukui potential value at covalent radius. For further details on the link between the Fukui potential and its link and performance as a descriptor of local hardness, the reader can refer to ref^53^.

The link between the Fukui potential and hardness suggests that the former is also a parameter related to the stability and reactivity of the chemical systems, namely, the lattice energy of inorganic ionic crystals. Hence, from eq 15, we propose the following model for the lattice energy in terms of the Fukui potential at the nuclei of the parent atoms that form a solid
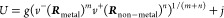
16

Note that eq 16 was derived for a 1:1 stoichiometry (MX). However, if one repeats the procedure from eqs 9 to 15 for a system with a *m/n* stoichiometry (M_*m*_X_*n*_), one concludes that powers *m* and *n* in eq 16 belong to the model. One can also resort to dimensional analysis to justify the powers in eq 16: the terms involving the products of the Fukui potential should have dimensions of energy. *g* and *j* are the constants taking different numerical values for different stoichiometries.

The approximations used to arrive at eq 15 are inherited by the model proposed in eq 16. Therefore, it is important to highlight and discuss them:

(i) The model assumes that the bonding is entirely covalent. This, in principle, introduces an error in systems where the bond has some covalent characteristic. However, the fact that eq 10 has polarization effects alleviates this difficulty somewhat since a charge transfer between atoms can be viewed as a large polarization of the electron density. Furthermore, the simplification of the linear response function (eq 12) may not be sufficient in cases where some degree of charge transfer occurs.
(ii) The model implies that all man–body interactions between atoms are electrostatic and captured by the Madelung constant. This, however, is an advantage of our model because it is only necessary to calculate the electronic properties of the molecule corresponding to the unit formula of the solid. Here, a degree of freedom is introduced into the model, which is the geometry of the molecule in which the Fukui potential is evaluated. Two alternatives are evident. One is to use the geometry corresponding to the position of the atoms in the solid and the other is to use the equilibrium geometry of the molecule in the gas phase. Although the first option allows us to partially introduce the ≪environment≫ of the atoms in the solid, it makes the model use information from the crystal structure of the solid. The second option, although it could lead to inaccuracy by not incorporating the information on the geometry of the solid, has the enormous advantage of simplifying the model and making it a simple tool for quickly scanning solids for which no information on the crystal structure is available. In the next section, we will see that this strategy is quite satisfactory.

To check the link between the Fukui potential and the lattice energy, we calculated the Fukui potentials at the nuclei of many simple inorganic ionic molecules, which constitutes the unit formulas of the corresponding ionic crystals, and checked for the performance of eq 16.

## 3 Computational Details

Geometry optimizations of all molecules in Tables 1, 2, and 3 were performed using DFT with the B3LYP exchange–correlation functional. This functional is accurate enough to predict the geometry of diatomic molecules. We have shown that a Popple triple-ζ basis set is flexible enough to compute the Fukui potential at the nucleus and other alchemical derivatives.^53,56^ However, here we used both 6-311+g (d,p) and def2-TZVPPD (at th) and found no significant differences. The Fukui potential was evaluated as the electrostatic potential of the Fukui function on each nucleus following the method discussed in refs^54^ and (56). However, a short discussion is in order. Note from eq 16 that the Fukui potential at nuclear positions is the electrostatic potential (at the nucleus) of charge distribution equal to the Fukui function. At zero temperature, the Fukui function is exactly given by
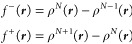
17

Hence, the Fukui potential can be written in terms of the electronic part of the molecular electrostatic potentials (MEP) of the neutral molecule (Φ) and its vertical ions (Φ^*N*–1^, Φ^*N*+1^)
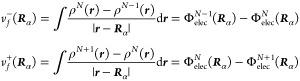
18

Note that because eq 17 is exact, not further approximation is introduced to eqs 15 and 17. In the case of the Gaussian basis set, the MEP can be computed analytically, and it is available in most electronic structure codes.

Note that it is not uncommon to find literature stating that eq 17 is a finite-difference approximation to the derivative of the electron density with respect to the number of electrons. In a seminal paper, Perdew, Parr, Levy, and Balduz^62^ showed the energy and the electron density of a molecular system at 0 K have derivative discontinuities and that eq 16 is exact.

The Fukui potential and chemical hardness were computed in a recent implementation of ChemTools,^58^ which is dedicated to computing chemical response functions. Other calculations were done with the *Gaussian 09* program.^59^

## 4 Results and Discussion

**Figure 1:**
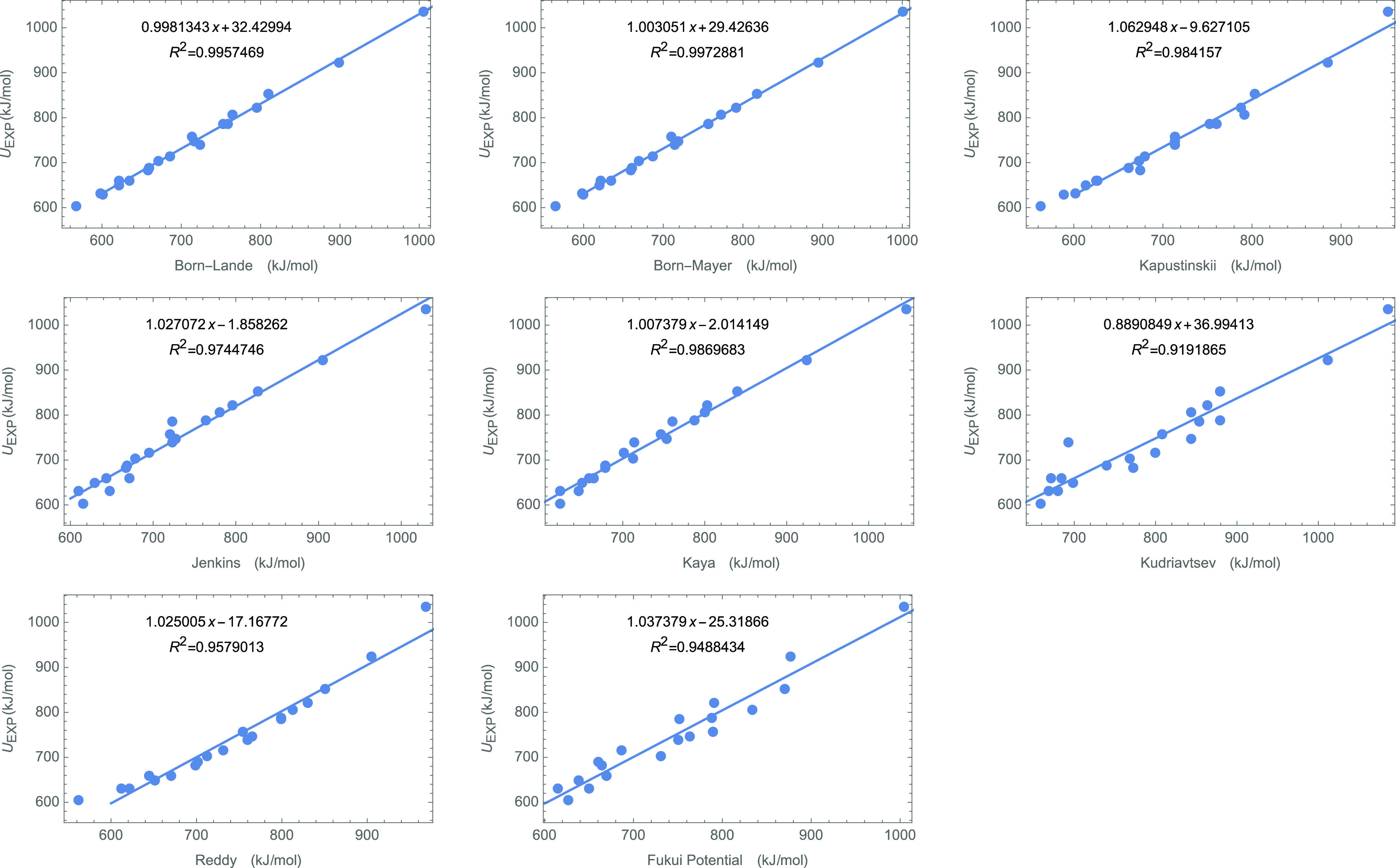
Correlation between experimental values of the lattice energy (in kJ/mol) of alkali halide solids and those predicted by different models. The last plot corresponds to the model based on the Fukui potential proposed in this work (eq 16). Each plot shows its linear fit and the correlation coefficient *R*^2^.

First, the results obtained by our model (eq 16) must be compared with the results from other models, such as those presented in the introduction and others available in the literature. Due to the limitation of the available data, only alkali halide crystals are considered (see Table 1).^63^ Born–Lande, Born–Mayer, and Kapustinskii equations are based on a purely electrostatic approach. Hence, it is not surprising that those methods correlate very well with experimental lattice energies in strongly ionic systems such as alkali halide (see Figure 1). VBT methods, such as the ones by Jenkins and Kaya, also perform very well in predicting the lattice energy of these systems (*R*^2^ = 0.974 and 0.987, respectively). Our proposal based on the Fukui potential of diatomic molecules does not perform worse (*R*^2^ = 0.949) than the other methods available, such as Reddy’s (*R*^2^ = 0.951) and Kudriavtsev’s (*R*^2^ = 0.919). It is important to note that the only information required to evaluate the lattice energy through eq 16 is the Fukui potential at the atoms of the parent molecule associated with the unit formula of the solid. Other methods require information on the crystal structure (Born–Lande, Born–Mayer, and Zhang) or the molar volume of the solid (Jenkins and Kaya). Hence, given the simplicity of eq 16, it surprises that it performs as well as methods that include the information of the structure of the solid.

**Table 1:** Comparison of the Lattice Energy Values (kJ/mol) Obtained via Various Theoretical and Experimental Approaches for Alkali Halides

alkali halides	Born–Haber–Fajans cycle (Exp)	Born–Lande	Born–Mayer	Kapustinskii	Jenkins	Kaya	Kudriavtsev^51^	Reddy^52^	Zhang	Equation 16
**LiF**	1036	1005	1000	952	1029	1046	1085	968	1032	1005
**LiCl**	853	810	818	803	827	839	880	851		870
**LiBr**	807	765	772	792	780	800	844	813		834
**LiI**	757	713	710	713	721	746		755		790
**NaF**	923	899	894	885	905	924	1011	905		877
**NaCl**	787	753	756	752	764	787	879	799	785	788
**NaBr**	747	717	719	713	727	753	844	765		763
**NaI**	704	671	670	673	678	713	768	712		731
**KF**	821	795	792	788	796	803	863	831		791
**KCI**	715	686	687	680	695	701	799	732		686
**KBr**	682	658	659	675	667	679	772	699		664
**KI**	649	622	620	613	630	650	699	651	647	639
**RbF**	785	758	756	760	723	761	853	799		752
**RbCl**	689	659	661	662	668	679	740	701	686	660
**RbBr**	660	634	635	626	644	659	685	670		669
**RbI**	630	601	600	589	610	623	680	622		615
**CsF**	740	724	714	713	723	714	693	760		750
**CsCl**	659	621	621	625	672	664	672	644		670
**CsBr**	631	598	598	602	648	646	669	612	630	650
**CsI**	604	568	565	563	616	623	659	562		627

From Table 1, it is not clear whether eq 16 has to over- or underestimate the lattice energy: the average error is only 2.4 kJ/mol. We know that the interatomic distance in the gas phase of the alkali halide molecules underestimates the interatomic distance in the solid by about 13%. We also know that the Fukui potential in the nucleus tends to decrease as the size of an atom decreases (see refs (51) and (51)). Therefore, when using the Fukui potential of gas-phase molecules, one would expect that the lattice energy would always be underestimated. Since this is not always the case, it can be said that the discrepancies between the model and the experimental data are due to a complete capture of polarization effects and to limitations of the model itself.

**Figure 2:**
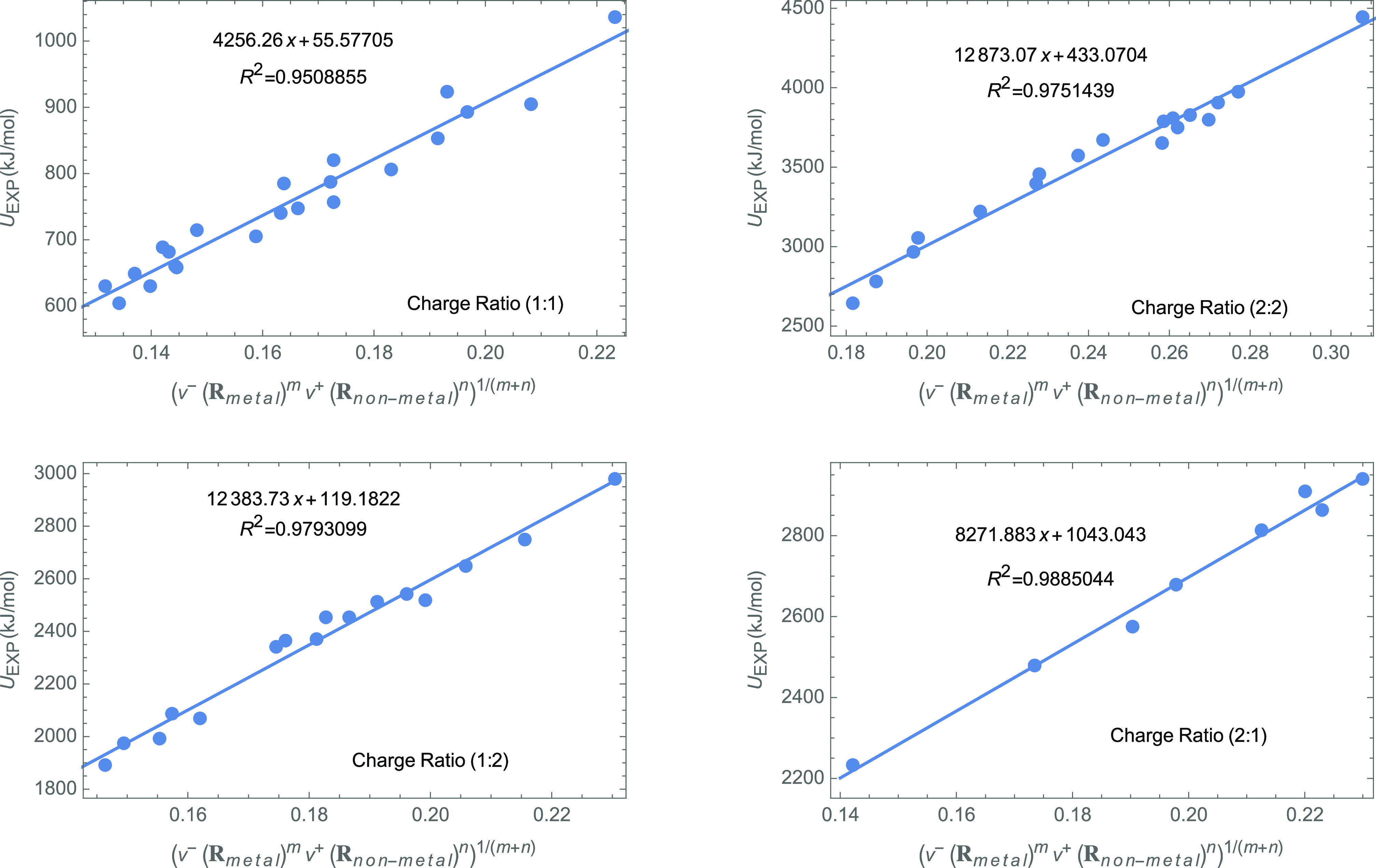
Correlation between the Fukui potential (in a.u.) and the lattice energy (in kJ/mol) of the inorganic ionic system for (top-left) MX type (charge ratio 1:1), (top-right) M_2_X_2_ type (charge ratio 2:2), (bottom-left) MX_2_ type (charge ratio 1:2), and (top-right) M_2_X_1_ type (charge ratio 2:1)

Having shown that for alkali halides our model is of no lesser quality than the models containing more information about the crystal structure of the material, it is time to evaluate the performance of the model in more diverse systems, including those where the covalent characteristic of the bond is not minor or negligible. Figure 2 shows the linear regressions of the experimental lattice energy and the product  for a set of systems (see Table 1) with stoichiometry **MX**, **M**_**2**_**X**_**2**_ (with a charge ratio of 2:2), **MX**_**2**_, and **M**_**2**_**X**, respectively. The performance of eq 16 is quite satisfactory in terms of the regression coefficient, ***R***^**2**^, which is greater than 0.97 for all cases but **MX** systems (*R*^2^ = 0.95). The constants *g* and *j* in eq 16 greatly depend on the stoichiometry of the solid (see in Table 2). This dependency is expected as *g* must include the information of the Madelung constant of the crystal. That *g* follows the same behavior as Madelung constants, that is, that g for **MX** and **M**_**2**_**X**_**2**_ are quite similar, suggests that *g* is an effective Madelung constant for the electrostatic interaction between distribution of charges equal to the Fukui potential.

**Table 2:** Constants of the Best Linear Fitting of Lattice Energies to eq 16

**crystal charge ratio**	***g***	***j***[kJ/mol]
**MX(1:1)**	4256.3	55.58
**MX**_**2**_**(2:1)**	12384	119.18
**M**_**2**_**X(1:2)**	8271.9	1043.0
**MX(2:2)**	12873	433.07

With the exception of the method by Zhang and the one proposed here, all methods explored here neglect any covalent characteristic of the crystal. In the case of Zhang, covalence is explicitly included in the model. Although in the derivation of eq 16 electron transfer between ions was neglected, it is the presence of the linear response function that can explain some degree of covalence in the bond. An electron transfer can always be thought of as an extreme polarization of the electron density over long distances. In our systems, the bond is clearly more ionic than covalent. Hence, this is a scenario in which approximations to the linear response function suffices to capture rearrangements of the electron density typical of partial covalent bonds.^60^ From Table 3, one can see that the Fukui-potential-based lattice energy equation provides quite close results to the data obtained via the Born–Haber–Fajans thermochemical cycle in crystals with partial covalent characteristic. Good examples of that are HgO, CdO, and Cu_2_S.

**Table 3:** Calculated Fukui Potential (*E*_h_), Ionization Energy (eV), Electron Affinity (eV), Hardness (eV), and Lattice Energies (kJ/mol) for Molecules That Correspond to the Unit Formula of Studied Solids

molecule/crystal	*v*^–^ (metal)	*v*^+^ (non-metal)	*I*	*A*	η	*U* form eq 16	Exp *U*
**LiF**	0,328	0,152	11,722	0,318	5,702	1005	1036
**LiCI**	0,258	0,142	10,072	0,583	4,744	870	853
**LiBr**	0,241	0,139	9,502	0,653	4,424	834	807
**LiI**	0,221	0,135	8,737	0,725	4,006	790	757
**NaF**	0,268	0,139	10,397	0,591	4,903	877	923
**NaCl**	0,223	0,133	9,330	0,787	4,271	788	787
**NaBr**	0,211	0,131	8,887	0,843	4,022	763	747
**NaI**	0,197	0,128	8,258	0,905	3,677	731	704
**KF**	0,249	0,120	9,827	0,406	4,711	791	821
**KCI**	0,193	0,114	8,754	0,635	4,060	686	715
**KBr**	0,183	0,112	8,337	0,696	3,820	664	682
**KI**	0,171	0,110	7,747	0,770	3,488	639	649
**RbF**	0,233	0,115	9,477	0,405	4,536	752	785
**RbCl**	0,182	0,111	8,545	0,628	3,959	660	689
**RbBr**	0,191	0,109	8,348	0,690	3,829	669	660
**RbI**	0,162	0,107	7,574	0,764	3,405	615	630
**CsF**	0,24	0,111	9,501	0,244	4,629	750	740
**CsCl**	0,197	0,106	8,608	0,479	4,064	670	659
**CsBr**	0,188	0,104	8,239	0,546	3,847	650	631
**CsI**	0,177	0,102	7,709	0,625	3,542	627	604
**AgBr**	0,233	0,186	9,675	1,568	4,054	941	905
**AgI**	0,216	0,179	9,045	1,559	3,743	892	892
**BeO**	0,405	0,234	10,153	2,225	3,964	4395	4444
**MgO**	0,320	0,209	7,845	1,890	2,978	3762	3791
**CaO**	0,309	0,167	7,035	0,823	3,106	3357	3401
**SrO**	0,288	0,158	6,636	0,701	2,967	3179	3223
**BaO**	0,281	0,139	6,798	0,392	3,203	2977	3054
**BeS**	0,318	0,221	9,237	2,343	3,447	3845	3832
**CaS**	0,237	0,163	7,832	2,091	2,870	2963	2966
**SrS**	0,225	0,156	6,962	1,302	2,830	2844	2779
**BaS**	0,237	0,139	6,669	1,153	2,758	2769	2643
**CoS**	0,291	0,229	6,624	0,855	2,885	3756	3653
**CuS**	0,291	0,250	8,674	2,825	2,924	3905	3795
**ZnS**	0,265	0,224	8,658	2,037	3,311	3569	3674
**CdS**	0,237	0,219	8,668	2,338	3,165	3365	3460
**HgS**	0,244	0,231	8,697	2,349	3,174	3489	3573
**MnO**	0,332	0,207	8,736	2,493	3,122	3807	3745
**ZnO**	0,313	0,245	6,956	2,823	2,066	3997	3971
**CdO**	0,285	0,239	9,152	2,248	3,452	3792	3806
**HgO**	0,285	0,260	8,688	2,234	3,227	3937	3907
**MgF**_**2**_	0,297	0,203	12,929	0,424	6,253	2973	2978
**MgCl**_**2**_	0,23	0,181	11,060	0,545	5,258	2547	2540
**MgBr**_**2**_	0,212	0,175	10,363	0,625	4,869	2429	2451
**MgI**_**2**_	0,191	0,167	9,448	0,676	4,386	2281	2340
**CaF**_**2**_	0,282	0,176	11,421	0,518	5,451	2669	2651
**CaCl**_**2**_	0,208	0,162	10,238	0,970	4,634	2299	2363
**CaI**_**2**_	0,176	0,149	8,941	1,196	3,872	2069	2087
**BaF**_**2**_	0,261	0,151	10,149	0,427	4,861	2363	2373
**BaCl**_**2**_	0,208	0,143	9,372	0,941	4,215	2125	2069
**BaBr**_**2**_	0,194	0,139	8,992	1,079	3,957	2042	1995
**BaI**_**2**_	0,177	0,133	8,440	1,235	3,603	1930	1890
**CdBr**_**2**_	0,206	0,196	10,294	0,896	4,699	2587	2517
**CdI**_**2**_	0,186	0,181	9,408	1,038	4,185	2381	2455
**SrF**_**2**_	0,26	0,164	10,700	0,608	5,046	2487	2513
**SrI**_**2**_	0,168	0,141	8,661	1,301	3,680	1970	1976
**ZnCl**_**2**_	0,238	0,205	11,284	0,247	5,519	2787	2748
**Li**_**2**_**O**	0,275	0,127	6,642	0,003	3,319	2801	2814
**Na**_**2**_**O**	0,223	0,105	5,072	0,271	2,401	2478	2478
**K**_**2**_**O**	0,210	0,065	4,398	0,155	2,121	2218	2232
**Cu**_**2**_**O**	0,255	0,187	7,947	1,190	3,379	2945	2939
**Ag**_**2**_**O**	0,242	0,182	8,265	1,359	3,453	2863	2910
**Cu**_**2**_**S**	0,232	0,206	7,836	1,230	3,303	2887	2865
**Ag**_**2**_**S**	0,209	0,177	7,663	1,328	3,168	2678	2677
**Tl**_**2**_**O**	0,213	0,152	7,523	0,003	3,760	2617	2575

**Figure 3:**
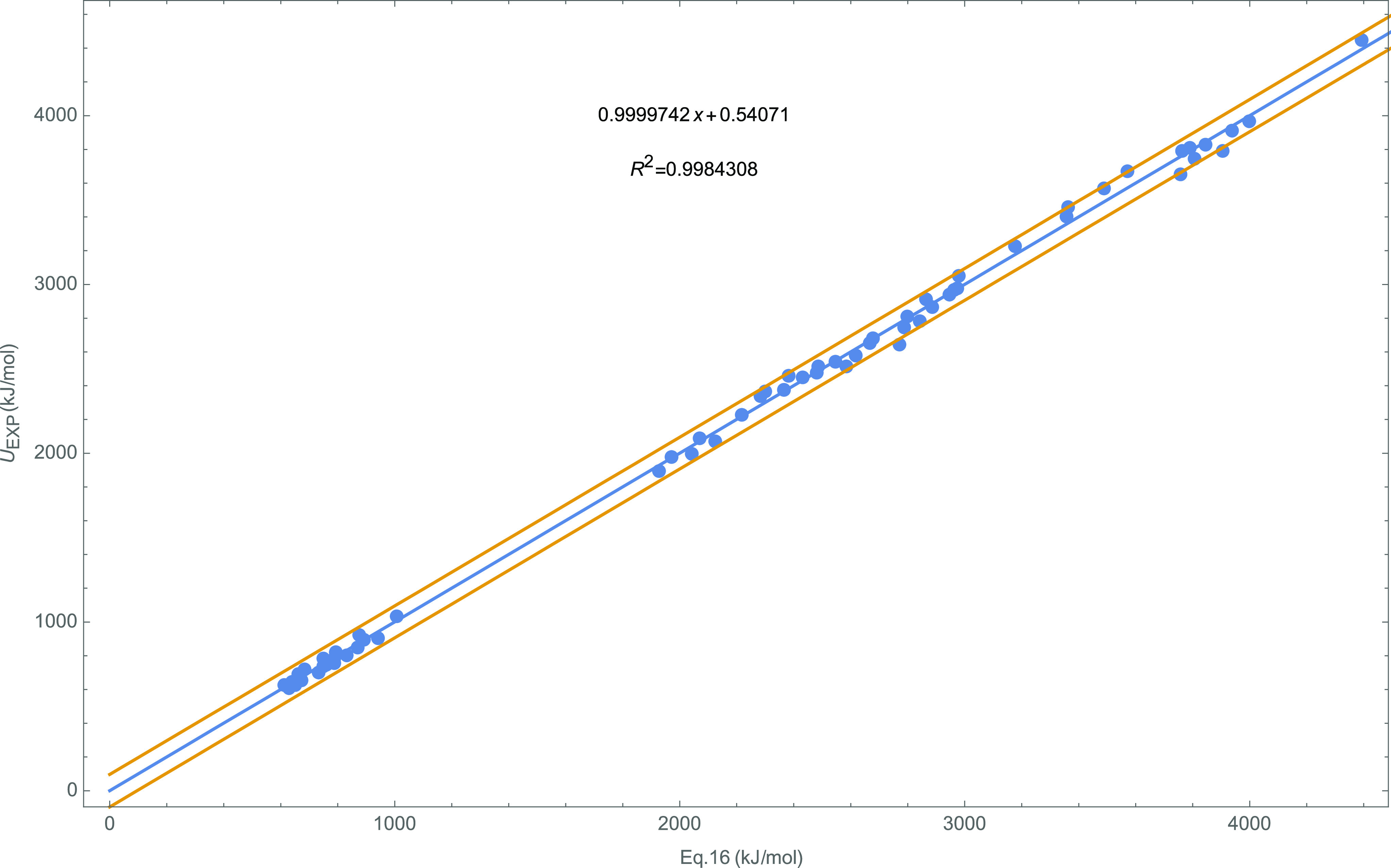
Correlation between the experimental values of the lattice energy (in kJ/mol) of solids in Table 3 the model based on the Fukui potential proposed in this work (eq 16). Orange lines are the confidence bands based on single observations (with a confidence of 95%).

Overall, the Fukui-potential-based lattice energy succeeds in correlating with the experimental values (those obtained with a Born–Haber–Fajans cycle) for all systems in Table 3 (see Figure 3). The correlation coefficient is surprisingly large (*R*^2^ = 0.99) if one takes into account that the energy values of the systems in Table 3 span over a large range of lattice energy, 604 < *U* < 4444 kJ/mol, and degree of covalence in the bonding. In order to rule out that this correlation is flawed, we made a 10-fold cross-validation test with the KNIME v4.5.1 package.^61^ This validation technique tests the linear regression against random samples taken from the data set. The test shows that the Fukui-potential-based model explains over the 99% of the variance of the experimental lattice energies. As a result of the analysis, the model statistics were calculated as MAE = 36.2, MSE = 2122.7, and RMSE = 47.0 kJ/mol.

**Figure 4:**
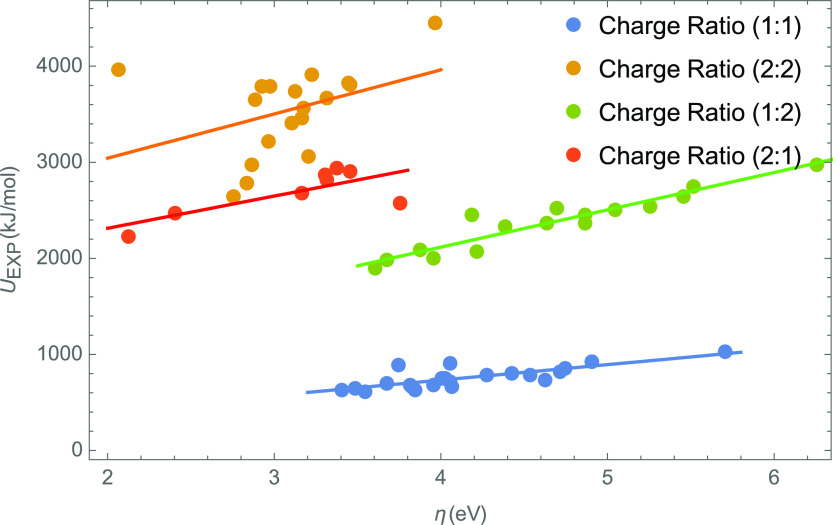
Lattice energy vs chemical hardness of inorganic ionic solids. **MX** type (charge ratio 1:1), **M**_**2**_**X**_**2**_ type (charge ratio 2:2), **MX**_**2**_ type (charge ratio 1:2), and **M**_**2**_**X**_**1**_ type (charge ratio 2:1).

The MHP states that hard molecules are thermodynamically more stable than soft molecules. In the solid state, Kaya highlighted the relationship between chemical hardness and thermodynamic stability through eq 7. However, this equation does not directly imply that inorganic solids meet the MHP because the molar volume and chemical hardness are not independent. In fact, it is well known that the hardness of atoms and ions decreases with radius.^42^Figure 4 shows a scatter plot of the lattice energy versus chemical hardness. For the whole set of solids, the lattice energy does not necessarily increase with hardness. However, when data is analyzed by a family of compounds, there seem to be a positive, yet weak, correlation between hardness and the lattice energy of compounds **MX**, **MX**_**2**_, and **M**_**2**_**X**. This case illustrates the limitations of the so-called chemical reactivity principles. Whereas in physics, a principle is unbreakable (the uncertainty principle), in chemical reactivity, principles are more guiding rules.^62^

## 5 Conclusions

In this work, we have developed a formal approach to the problem of determining the lattice energy of inorganic solids using conceptual DFT tools. In particular, we have shown that the lattice energy has a lower bound determined by the Fukui potential (eq 15). This leads us to postulate an ansatz for the lattice energy in terms of the Fukui potential in the nuclei (eq 16). Our model is tested against experimental data for a series of inorganic solids and also checked against other models in available in the literature. The results show that, within the systems studied, the Fukui potential is as good a descriptor as the other models. Our model has, however, the following advantages: (i) it is entirely supported by DFT perturbation theory and is written in terms of well-known reactivity descriptors. (ii) For its evaluation, it is not mandatory to know information about the crystalline structure of the solid. (iii) Only an ab initio calculation on the molecule of the unit formula of the solid is required to estimate the lattice energy. (iv) The combination of (ii) and (iii) makes this method easy to apply in the scanning of large libraries of materials.

We also assessed the validity of MHP in this type of solids, observing that this principle is quite limited. Only a weak correlation is observed between lattice energy and chemical hardness. Although the quality of correlation depends on the type of systems, we believe that this is a case that reveals that the so-called principles of reactivity are guiding rules and not principles in a physical sense.

## Supporting Information Available

Results of n-fold cross-validation and Bland–Altman diagram (PDF)
