# Supplementary material for: On the Prediction of Lattice Energy with the Fukui Potential: Some Supports on Hardness Maximization in Inorganic Solids
Source: J Phys Chem A. 2022 Jun 29;126(27):4507–16. doi: 10.1021/acs.jpca.1c09898 (PMC9289887; doi:10.1021/acs.jpca.1c09898)
Supplement: Supplementary file 1 — jp1c09898_si_001.pdf [file jp1c09898_si_001.pdf]

Supplementary File

On the Prediction of Lattice Energy with the Fukui Potential: Some Supports on Hardness Maximization in Inorganic Solids

Savaş Kaya,[1,*] Andrés Robles-Navarro[2], Erica Mejía[3], Tatiana Gómez[4*], Carlos Cardenas[2,5*]

[1]Sivas Cumhuriyet University, Health Services Vocational School, Department of Pharmacy, 58140, Sivas/Turkey

[2]Departamento de Física, Facultad de Ciencias, Universidad de Chile, Las Palmeras 3425, Santiago Casilla 653, Chile

[3] Institución Universitaria Pascual Bravo – Facultad de Ingeniería– (Medellin-Colombia)

[4]Theoretical and Computational Chemistry Center, Institute of Applied Chemical Sciences, Faculty of Engineering, Universidad Autonoma de Chile, Santiago, Chile

[5]Centro para el Desarrollo de la Nanociencia y la Nanotecnología (CEDENNA), Avda. Ecuador 3493, Santiago 9170124, Chile

savaskaya@cumhuriyet.edu.tr

cardena@uchile.cl

tatiana.gomez@uautonoma.cl

The consistency of the experimental and theoretical results was examined by the cross validation method. In order to show that this analysis gives accurate results at different levels, cross validation analyzes with 4, 5, 8, 10, 12 and 16 parts (fold) were carried out, taking into account the number of n. Adjusted (Adj.) R2, Mean Square Error (MSE), Root Mean Square Error (RMSE) statistics are included in the evaluation of the analysis results. The results are presented in Table S1.

Table S1: The results of n-Fold Cross Validation Results

n-Fold	Adj. R^2	MSE	RMSE
4	0,997	2545,16	50,45
5	0,997	2240,88	47,34
8	0,997	2176,88	46,66
10	0,997	2254,65	47,48

12	0,997	2215,85	47,07
16	0,997	2285,91	47,81

When the results in the table are checked, it is seen that Adj. R^2 values are calculated very close to 1 at all n-Fold levels. Similarly, with the RMSE statistic being calculated as a minimum of 46.66 and a maximum of 50.45, it is understood that the difference between the experimental and calculated values is quite small. In addition, Bland-Altman analysis was applied to see the reliability of new derived lattice energy equation. Figure S1 visualizes the results obtained results via Bland-Altman analysis

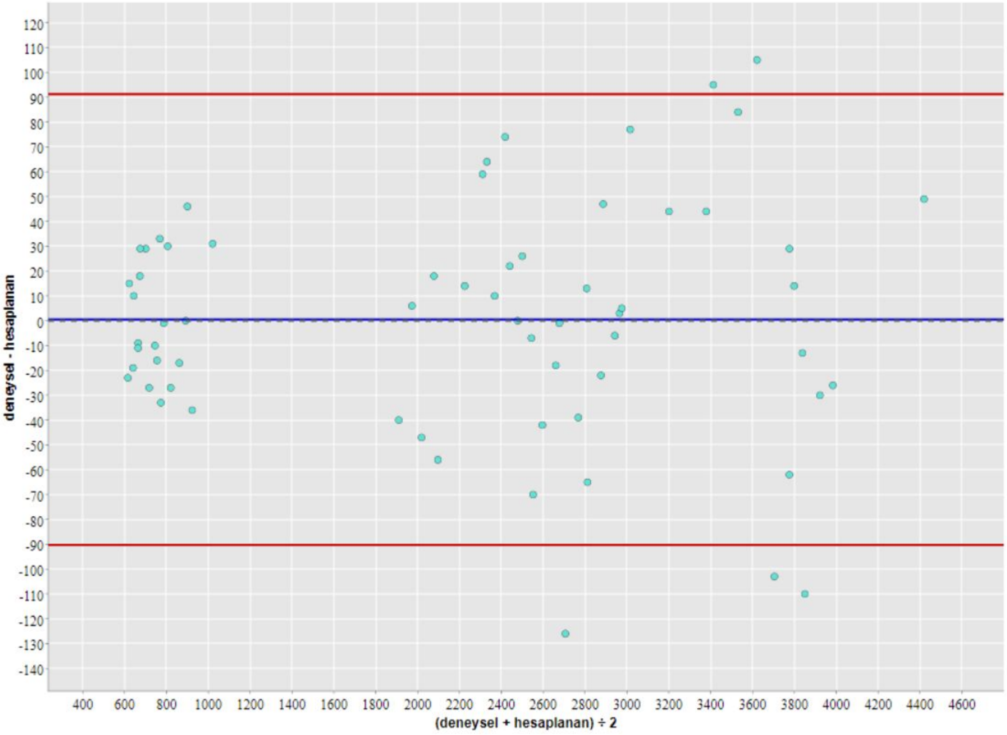

Figure S1: Bland-Altman diagram obtained for this study.

According to the results of the analysis, the bias between the two variables was calculated as 0.484. At the same time, the upper acceptable limit was calculated as 91,219 and the lower acceptable limit as -90.25. To illustrate, the limit of difference between measurements between experiment and calculation has been calculated to be approximately 90 (according to both negative and positive magnitudes). As seen be seen from the diagram, there are only 5 values exceeding this limit (2 measurements exceeding the upper limit and 3 measurements exceeding the lower limit). From this point of view, it would not be wrong to state the estimation success

of the model used as 95.31% (due to the ratio of 61/64). The success of the model established according to both n-Fold Cross Validation and Bland-Altman results was calculated over 95%.
